# 

**Published:** 1887-09

**Authors:** 


### OUR ADVERTISERS.

ALWAYS read the advertisements; you will find something to interest and benefit you.

FAIRCHILD BRO. & FOSTER present some new and interesting facts to our readers this month through their new ad. to be found on page 12.

MILLER'S DRUG CO., ST. LOUIS.—See new advertisement of this company in this issue. The company does a big business in instruments as well as drugs. Write for their new reduced price-list of instruments. It will be mailed free on application.

TARRANT & CO., NEW YORK.—See advertisement of this old and established house, to be found on the first advertising page of THE JOURNAL. They have earned an enviable reputation for their goods all over the country. Refer to the advertisement and write them for any information you may desire.

SMITH & BRADFIELD, ATLANTA.—This firm has a very attractive advertisement in this issue of THE JOURNAL, to which we desire to call the attention of our readers. These gentlemen carry on the largest wholesale drug business in Georgia and make a specialty in furnishing physicians' supplies. Those of our readers who have to carry their own stock of drugs will find it to their interest to communicate with this firm. Their manufacturing department is complete, and their goods are all fully up to the U. S. D. standard. See ad. and write them for prices.

NERVOUS PROSTRATION, DYSMENORRHŒA.—Dr. N. A. Sackett, Ewing, Nebraska, says: "I have tested celerina in two cases of nervous prostration. One case was a man of about 35 years of age, who has been subject to attacks for a number of years as often as every two weeks. I prescribed an ounce in two ounces of port wine, to take a teaspoonful four times a day. He has not had an attack since, although two months have elapsed. The other was a lady of about the same age, who has had similar attacks for the last five years. She has had no recurrence of the trouble since; and moreover she has passed two monthly periods without the usual dysmenorrhœa, with which she is usually afflicted at that period. I shall continue to prescribe it in cases in which it is indicated, and will report more fully in the future."

